# Repeated Solid-state Dewetting of Thin Gold Films for Nanogap-rich Plasmonic Nanoislands

**DOI:** 10.1038/srep14790

**Published:** 2015-10-15

**Authors:** Minhee Kang, Sang-Gil Park, Ki-Hun Jeong

**Affiliations:** 1 Korea Advanced Institute of Science and Technology (KAIST), Department of Bio and Brain Engineering and KAIST Institute for Optical Science and Technology, 291 Daehak-ro, Yuseong-gu, Daejeon 305-701, Republic of Korea

## Abstract

This work reports a facile wafer-level fabrication for nanogap-rich gold nanoislands for highly sensitive surface enhanced Raman scattering (SERS) by repeating solid-state thermal dewetting of thin gold film. The method provides enlarged gold nanoislands with small gap spacing, which increase the number of electromagnetic hotspots and thus enhance the extinction intensity as well as the tunability for plasmon resonance wavelength. The plasmonic nanoislands from repeated dewetting substantially increase SERS enhancement factor over one order-of-magnitude higher than those from a single-step dewetting process and they allow ultrasensitive SERS detection of a neurotransmitter with extremely low Raman activity. This simple method provides many opportunities for engineering plasmonics for ultrasensitive detection and highly efficient photon collection.

Spatial localization of intense electromagnetic (EM) fields near the nanostructures termed in ‘hotspots’^1^,^2^ can be created by strong coupling of plasmonic near-fields between neighboring metallic nanostructures. The hot spots bring about the extraordinary increase of Raman scattering from neighboring molecules, which enables the ultrasensitive detection of biochemical molecules without fluorescent labeling, *i.e.*, surface enhanced Raman scattering (SERS)^3^. Recently, multiple hotspots between diverse plasmonic nanostructures advance the SERS detection of small biomolecules at low concentrations and even at a single-molecule level^4^,^5^. However, this ultrasensitive detection still faces a problem with the reproducibility of plasmonic nanostructures for stable Raman signal enhancement^6^,^7^ and thus the construction of nanogap-rich SERS-active nanostructures still remains challenging. Based on the site enhancement in SERS, highly active SERS sites account for only 63 sites in 1,000,000 of the total sites but contribute to 24% of the overall SERS intensity^8^.

High yield nanofabrication of nanogap-rich SERS substrates such as randomly dispersed nanoparticles^9^, nanometer scaled rough film or well-defined nanostructures via e-beam lithography (EBL)^10^, or nanosphere lithography (NSL) and ion etching^11^, has attracted substantial interest over the last decade. More recently, the combinations of nanolithographic patterning with templated growth or high aspect ratio negative resist process^12^,^13^ enable the nanofabrication of sub-nanogap metal nanostructures whereas these methods still have technical challenges such as low cost, large area fabrication, and tuning capability of plasmon resonance wavelength. Lithography-free nanofabrications by using self-assembled metal nanoparticles in solution phase can also overcome such limitations with large-area homogeneity in a highly reproducible manner^14^,^15^. However, additional chemical modification and other driving forces such as electrostatic and hydrodynamic interaction are still required for assembling metallic nanoparticles on a substrate^16^,^17^. Alternatively, solid-state dewetting of metal thin films^18^ has distinctive advantages for producing metal nanoisland arrays over a large area with enhancement factor over 10^5^ 15,19. The average size and separation of metal nanoislands can be moderately controlled with the initial thickness of a thin metal film^20^,^21^. However, the concurrent fabrication of small gap spacing and large nanoisland size still remains challenging: as the film thickness increases, both the average nanoisland size and the interstitial gap spacing simultaneously increase after a single-step solid-state dewetting process (see Fig. S1 in supplementary information). This unique nature intrinsically confines the use of solid-state metal dewetting in fabricating highly scattered SERS substrates, *i.e.*, large nanoislands with small gap spacing. Moreover, this trade-off between the size and the gap hinders the precise tuning of plasmon resonance wavelength because the plasmon resonance typically becomes red-shifted as the nanoisland size increases but blue-shifted as the gap spacing increases^22^,^23^. Very recently, multiple dewetting allows the size control of nanoislands^24^, however, the quantitative analysis is still in need for the size-gap control and plasmon resonance tuning as a practical guideline for developing engineered plasmonic nanostructures.

Here, we report nanogap-rich gold nanoislands with enlarged scales for highly sensitive SERS substrates by using simply repeating solid-state dewetting of thin Au film. The schematic illustration compares two different Au nanoislands created by using a single and repeated solid-state dewetting (Fig. 1a,b). Unlike a single-step dewetting, the repeated dewetting can provide enlarged Au nanoislands with small gap spacing, which increase the number of plasmonic hotspots and thus enhance the extinction intensity as well as the tunability for plasmon resonance wavelength. The plasmon resonance was numerically calculated by using a finite-difference time-domain (FDTD) method, where the Au nanoisland was modeled as nanoisland arrays in a square lattice with a period of 100 nm. As the nanoisland diameter increases, the gap spacing between neighboring nanoislands decreases simultaneously. The calculated results clearly indicate that a substantial increase of the hotspots owing to the enlarged size with small gap spacing apparently contributes to an increase of extinction intensity as well as a substantial red-shift of plasmon resonance wavelength. (Fig. 1c).

## Results and Discussion

**Figure 2:**
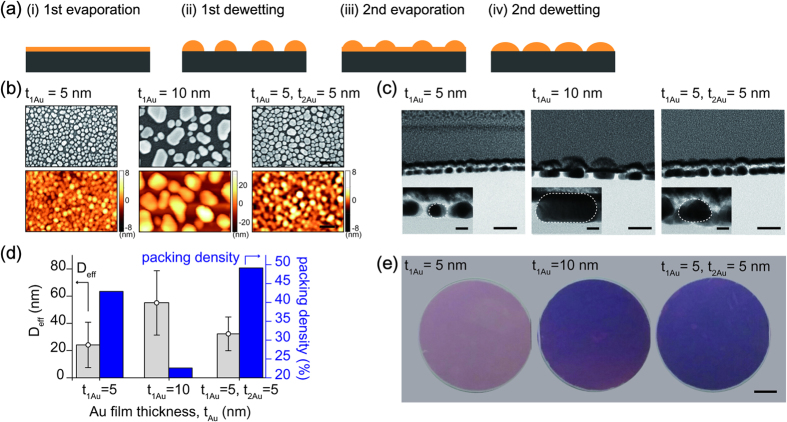
Nanofabrication procedures for repeated solid-state dewetting of thin Au film. (**a**) the nanofabrication steps of nanogap-rich Au nanoislands on a quartz substrate; (i) an initial thin Au film was thermally evaporated on a quartz glass wafer, (ii) the thin Au film was transformed into Au nanoislands after the first dewetting process, (iii) an additional thin Au film was thermally evaporated over the predetermined Au nanoislands, and (iv) subsequently annealed for enlarged Au nanoislands with small gap spacing. (**b**) SEM and AFM images of Au nanoislands from a single dewetting of 5 nm and 10 nm in initial film thickness and repeated dewetting of thin Au film with 5 nm in both and additional thickness, denoted by t_1Au_ = 5 nm, t_1Au_ = 10 nm for a single dewetting process and t_1Au_ = 5, t_2Au_ = 5 nm for the repeated dewetting process, respectively. (**c**) side-view TEM images of Au nanoislands with enlarged view of a single nanoisland. (**d**) the extracted effective diameters and packing densities of Au nanoislands from SEM images and (**e**) optical images of nanofabricated 4 inch wafers corresponding to a single and repeated dewetting process. (SEM and AFM image scale bar: 100 nm, TEM images: 50 nm and inset of TEM images: 10 nm).

Nanogap-rich Au nanoislands were simply achieved by alternating thin Au film evaporation and thermal annealing. In the first step, a thin Au film was thermally evaporated on a 4 inch quartz substrate and then thermally annealed to form Au nanoislands on a hot plate for one hour at 500 °C. In the second step, the second thin Au film was thermally evaporated on the pre-determined Au nanoislands and subsequently annealed at the same condition (Fig. 2a). This second step not only reduces the gap spacing between neighboring Au nanoislands but also increases the nanoisland size. A thin Au film on the Volmer-Weber mode^25^ of thin film growth initially forms isolated nanoislands, nucleates, and coalesce each other during the first step. However, the pre-determined nanoislands during the second step accelerates the rapid coalescence of nanoislands rather than the nucleation. Consequently, the second dewetting process enlarges the size of initially formed Au nanoislands as well as reduces the interstitial gap spacing between neighboring Au nanoislands. In this experiment, the repeated solid-state dewetting was consecutively performed from thermal evaporation and dewetting of additional 5 nm thick Au film after thermal dewetting of Au film with 5 nm in initial thickness (denoted by t_1Au_ = 5 nm, t_2Au_ = 5 nm). For comparison, Au nanoislands after a single-step was also obtained by using Au film with 5 nm (denoted by t_1Au_ = 5 nm) and 10 nm (denoted by t_1Au_ = 10 nm) in initial thickness, respectively. The SEM and AFM images show Au nanoislands under different dewetting conditions, *i.e.*, t_1Au_ = 5 nm, t_1Au_ = 5 nm, t_2Au_ = 5 nm and t_1Au_ = 10 nm, shown in Fig. 2b. Both the microscopic images apparently demonstrate enlarged Au nanoislands with small gap spacing after the repeating solid-state dewetting of thin Au film, compared to those after the single-step. Besides, the side view TEM images of Au nanoislands clearly support the enlarged size with small gap spacing (Fig. 2c). The effective diameter and packing density of Au nanoislands were calculated from the binary segmentation of Au nanoislands shown in the SEM images, where the error bar indicates the size distribution of Au nanoislands (Fig. 2d). This repeated solid-state dewetting process clearly exhibits both the high packing density from the enlarged effective diameter with small gap spacing and the relatively small size distribution, compared to a single-step solid-state dewetting, *i.e.*, Au nanoislands from Au film with 5 nm (t_1Au_ = 5 nm) and 10 nm (t_1Au_ = 10 nm) in initial thickness. The optical images also demonstrate a wafer-level fabrication of enlarged Au nanoislands with small gap spacing (Fig. 2e).

**Figure 3:**
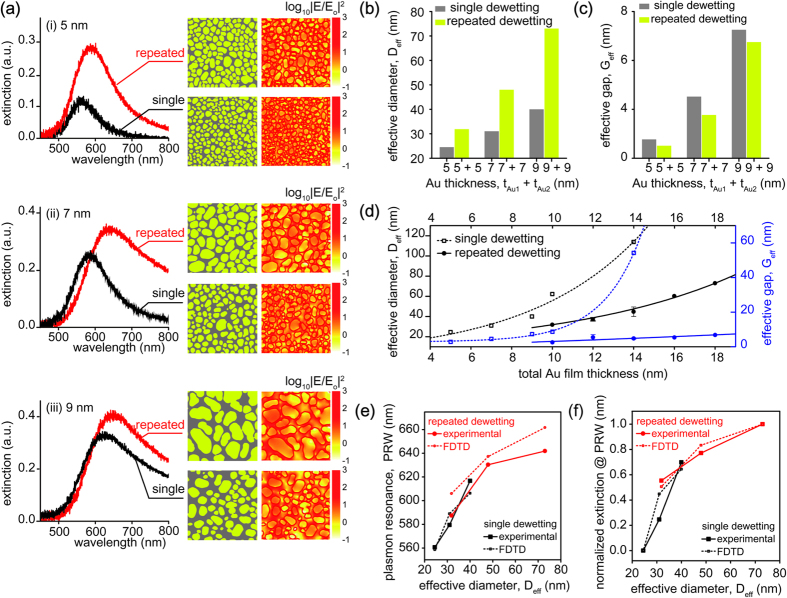
Tuning capabilities of Au nanoislands by using the repeated dewetting process. (**a**) Extinction spectra of a single-step and repeated solid-state dewetting of thin Au film with (i) 5 nm, (ii) 7 nm, and (iii) 9 nm in thickness. Binary segments of Au nanoislands from the SEM images (left) and numerically calculated electric field intensity distributions at the plasmon resonance wavelength from the SEM images by using a three-dimensional finite difference time domain (3D-FDTD) method (right). (**b**) the effective diameters and (**c**) the effective gaps after a single-step and repeated solid-state dewetting processes. (**d**) the effective diameter and the gap depending on total Au film thickness. The black and blue lines indicate a single-step and repeated dewetting, respectively. The 3D-FDTD calculated and experimental (**e**) plasmon resonace wavelengths and (**f**) corresponding normalized extinctions of Au nanoislands after a single-step and double dewetting, *i.e.*, repeated dewetting with the same thickness, depending on the effective diameter. The black and red lines indicate a single-step and repeated dewetting, respectively.

The enlarged Au nanoislands with small gap spacing apparently increase the number of plasmonic hotspots and thus enhance the extinction intensity as well as the tunability for plasmon resonance wavelength. The size and interstitial gap spacing of pre-determined Au nanoislands can be moderately controlled by the initial Au film thickness. The effective diameters of Au nanoislands from 5, 7, and 9 nm in initial thickness during the first dewetting process are about 25 nm, 31 nm, and 41 nm, respectively. In the first dewetting process, the gap spacing between neighboring Au nanoislands inherently increases with the nanoisland size, which also increases with the initial film thickness. In particular, an increase of the film thickness reduces the nucleated void density and widens the void opening during dewetting to reach a stable configurations, which consequently increases the gap spacing between Au nanoislands^26^,^27^. However, the repeated solid-state dewetting significantly surpresses an increase of the gap spacing whereas the nanoisland size becomes enlarged. This geometrical variation of Au nanoislands favorably contributes to a substantial red-shift of the plasmon resonance wavelength (PRW). For different Au film thicknesses of 5 nm, 7 nm, and 9 nm, the extinction spectra from a single-step (black line) and repeated dewetting (red line) clearly demonstrate that enlarged nanoislands with small gap spacing after repeated dewetting enhances both the extinction intensity and the red-shift of PRW (Fig. 3a). The experimental results also include the binary segments of Au nanoislands from the SEM images (left) and the electric field intensity distributions at their corresponding plasmon resonance wavelengths were numerically calculated by using a finite-difference time-domain (FDTD) method (right) (see Fig. S2. in supplementary information). A decrease in gap spacing results in stronger electromagnetic hotspots. The effective diameter and gap of Au nanoislands after a single-step and repeated dewetting were calculated from the binary segmented SEM images for different film thicknesses (Fig. 3b,c, Fig. S3 in Supplementary Information). The results clearly exhibit that the nanoisland size increases by more than twice but the gap spacing relatively remains constant after the repeated dewetting with double film thickness for different film thicknesses. In addition, based on the comparison between a single-step (dashed line) and repeated (solid line) solid-state dewetting, the effective diameter increases but the gap spacing remain constant as the total Au film thickness increases and therefore it concludes that the repeated solid-state dewetting enables the fabrication of small gap spacing Au nanoislands (Fig. 3d). Depending on the effective diameter, the calculated PRW (Fig. S4 in supplementary information) and normalized extinction intensity well agree with the experimental results, which indicate that both the PRW shift and the extinction values increases with the effective diameter (Fig. 3e,f). If a given Au thickness for the first dewetting process is thicker than additional film for the second dewetting process, the thinner additional film for the second dewetting process may give rise to multiscale nanoislands (Fig. S5 in supplementary information). This scaling behavior of the plasmon resonance coupling between Au nanoislands is because the plasmon coupling strength varies universally as a function of the size and gap spacing. Thus the repeated dewetting enables precise tuning of the PRW over a wide wavelength range by changing the size and gap spacing.

**Figure 4:**
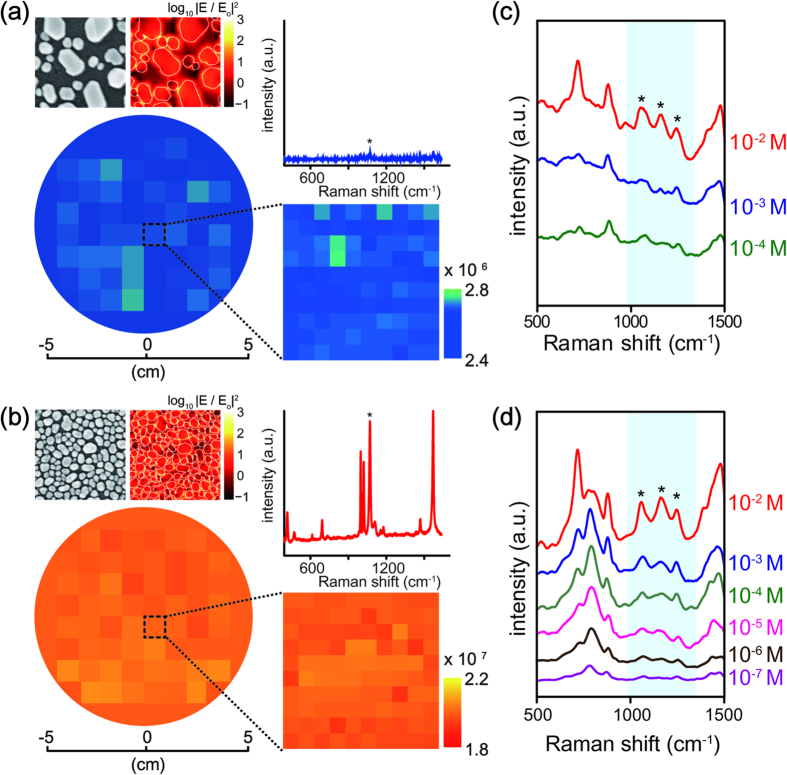
SERS enhancement factors (EF) of Au nanoislands and their SERS limit-of-detection. **a**,**b**) Spatial distribution of SERS enhancement factors (EFs) at ~1069 cm^−1^ of benzenethiol (BT) molecules at an excitation laser of 633 nm over an area of 4-inch glass substrate for (**a**) a single-step solid-state dewetting with t_1Au_ = 10 nm and (**b**) a repeated dewetting with t_1Au_ = 5, t_2Au_ = 5 nm. A expanded spatial map of SERS EFs at ~1069 cm^−1^ were acquired at 1 mm intervals over an area of 1 cm^2^ and has spatially averaged SERS EFs about ~10^6^ for 10 nm and ~10^7^ for t_1Au_ = 5 and t_2Au_ = 5 nm, demonstrating the excellent uniformity over a 4-inch substrate. The SEM images and numerically calculated electric field intensity distributions at excitation wavelength of 633 nm by using a three-dimensional finite difference time domain (3D-FDTD) method (upper left) and representative SERS spectra of BT molecules (upper right). (c-d) SERS limit-of-detection (LOD) of octopamine molecules with different concentrations. The LOD exhibits ca. 100 μM for (**c**) a single-step solid-state dewetting with t_1Au_ = 10 nm but increases down to 10 μM for (d) repeated dewetting with t_1Au_ = 5, t_2Au_ = 5 nm.

Highly dense EM hotspots and PRW matching of Au nanoislands are very crucial in achieving enhanced Raman signals from the plasmonic nanostructures^23^. In this experiments, SERS signals of benzenethiol (BT) molecules under an excitation laser of 633 nm were measured from two different Au nanoislands with a same total Au film thickness, *i.e.*, Au nanoislands after a single-step dewetting of 10 nm in initial thickness (t_1Au_ = 10 nm) and after repeated dewetting with 5 nm in both initial and additional thickness (t_1Au_ = 5 nm, t_2Au_ = 5 nm), respectively. The result shows the SEM images, the calculated electric field intensity distributions at an excitation wavelength of 633 nm (upper left), and representative SERS spectra of BT molecules (upper right) (Fig. 4a,b). In this experiment, the SERS signals at 1069 cm^−1^ of BT molecules were quantitatively measured under 633 nm excitation and the corresponding SERS enhancement factor (EF), *i.e.*, the average signal enhancement from absorbed molecules participated in SERS to that in Raman scattering, was spatially mapped with 1 cm intervals over two different 4 inch substrates. An expanded spatial map of SERS EF was also acquired at 1 mm intervals over an area of 1 cm^2^. The experimental results clearly demonstrate a substantial increase of SERS EF for Au nanoislands after repeated dewetting. Such substantial increase in the local field enhancement near EM hotspots can be explained by the small gap spacing between neighboring nanoislands^28^, which decreases by about 3.5 times compared to the gap spacing of a single-step dewetting. The effective area of SERS active sites, *i.e.*, surface coverage in SERS, also increases by about 2.2 times compared to that of a single-step dewetting.

Consequently, the averaged SERS EF from Au nanoislands after repeated dewetting increases by about 7.5 times (see below method of *SERS enhancement factor (EF)*) and thus the SERS intensity of BT molecules substantially increases by about 16.4 times. The limit-of-detection (LOD) in SERS with extremely low Raman activity molecule, *i.e.*, octopamine, which is a well-established neuromodulator with neurotransmitter functions that mediate diverse physiological process in the central nervous system (CNS)^29^ and plays an important role in different disease states, including hepatic encephalopathy^30^, schizophrenia^31^, and renal disease^32^, is ca. 100 μM at the signature peaks from Au nanoislands from a single-step dewetting process, however, the LOD for repeated dewetting is increased by one order of magnitude, *i.e.*, ca. 10 μM.

To be concluded, this work reports a simple and wafer-level fabrication for nanogap-rich metal nanoislands with enlarged sizes for highly sensitive SERS substrates by repeated solid-state dewetting of thin Au film. The precise control of the initial and additional Au film thicknesses enables manipulation of the size, interstitial gap spacing, packing density and plasmonic properties of Au nanoislands. The experimental results clearly demonstrate that Au nanoislands from repeated dewetting increases the SERS EF by about 7.5 times, compared to those from the single-step dewetting process. This repeated dewetting process can provide many opportunities for not only engineering plasmonics for ultrasensitive detection and highly efficient photon collection but also for nanoscale metal masks for reactive ion etching or seeds for the catalytic growth of nanowires.

## Methods

### Extinction measurement

The extinction spectra were measured with a microscopic spectrometer,which consists of an inverted microscope (Carl Zeiss Axiovert 200M) equipped with an excitation laser, and a spectrometer (MicroSpec 2300i) with a charge-coupled device (CCD) camera (Princeton Instruments, Model PIXIS: 400BR). The extinction spectra of silver nanoislands were collected with 50× lens (NA = 0.5).

### SERS measurement

A helium–neon laser (632.8 nm) and a spectrometer (MicroSpec 2300i spectrometer equipped with a charge-coupled device camera (Model PIXIS: 400BR, Princeton Instruments)) were coupled with an inverted microscope (Axiovert 200M, Zeiss). The excitation and collection of light were done by a 50× objective lens (NA 0.5). The excitation laser power was 5.15 mW and the acquisition time was 1 second.

### SERS enhancement factor (EF)

SERS EF was calculated by the conventional method, where the average enhancement factor of *EF*_*ave*_ is defined as 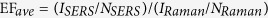, where *I*_*SERS*_ is the SERS intensity, *I*_*Raman*_ is the Raman intensity, *N*_*SERS*_ is the number of probed molecules in SERS, and *N*_*Raman*_ is the number of probed molecules in Raman measurements. The intensity of the 1069 cm^−1^ peak of BT molecules from the SERS and Raman measurements have been used for SERS EF calculation. The effective SERS active surface area of repeated dewetting from Au film of 5 nm in both initial and additional thicknesses is increased by ca. 2.2 times, compared to that from a single-step dewetting of 10 nm thick Au film. The surface coverage of benzenthiol self-assembled on gold is about 6.8*10^14^/cm^−2^ from the literatures^33^,^34^. SERS intensity (*I*_*SERS*_) over the number of probed molecules in SERS (*N*_*SERS*_) is about 9.9*10^−7^ for a single-step of solid-state dewetting of 10 nm thick Au film and 7.4*10^−6^ for repeated dewetting from Au film of 5 nm in both initial and additional thicknesses. The number of BT molecules (*N*_*Raman*_) was calculated from the average number of molecules in the detection volume. The detection beam spot radius is about 2.5 μm and experimentally measured collection depth is about 110 μm. The density of BT molecules is 1.073 g/ml, and the molecular weight is 110.18 g/mol. Therefore, the number of BT molecules (*N*_*Raman*_) contributing to the normal Raman signal measured from the standard is 1.3*10^13^ and the Raman intensity (*I*_*Raman*_) over the number of probed molecules in Raman measurements (*N*_*Raman*_) is 3.7*10^−13^. From the calculation results described above, *EF*_*ave*_ is about 2.7*10^6^ for a single-step dewetting of 10 nm thick Au film and 2.0*10^7^ for repeated dewetting from Au film of 5 nm in both initial and additional thicknesses.

### Numerical analysis of E-field distribution

The electric fields of Au nanoislands from different initial and additional thicknesses were numerically calculated by using a three-dimensional finite-difference time-domain method (Lumerical FDTD Solutions 8.0.6). The geometric configuration of Au nanoislands were extracted from the binary segmented SEM images by using Image J software and the individual Au nanoislands were considered as a cylindrical shape (see Fig. S2 in supplementary information).

## Additional Information

**How to cite this article**: Kang, M. *et al.* Repeated Solid-state Dewetting of Thin Gold Films for Nanogap-rich Plasmonic Nanoislands. *Sci. Rep.* **5**, 14790; doi: 10.1038/srep14790 (2015).

## Supplementary Material

Supplementary Information

## Figures and Tables

**Figure 1:**
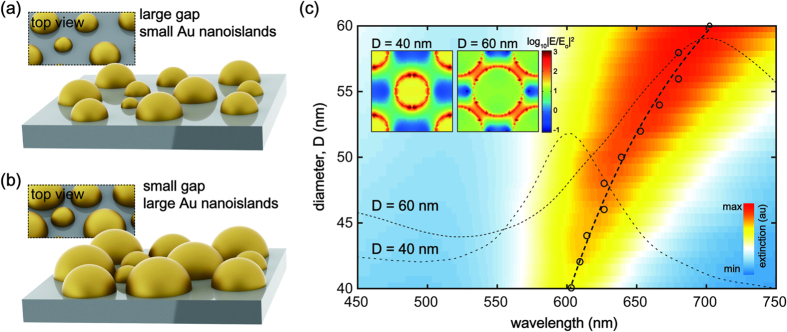
Au nanoislands and plasmon resonance shift depending on the diameter. A schematic illustration for Au nanoislands from (**a**) a single-step solid-state dewetting and (**b**) highly dense and nanogap-rich Au nanoislands after repeated dewetting. Unlike a single-step solid-state dewetting of thin Au film, the repeated dewetting simply provides Au nanoislands with enlarged size but small gap spacing, which increase the packing density for strongly coupled and multiple electromagnetic hotspots. (**c**) the extinction and plasmon resonance shift depending on the diameter of Au nanoislands; the dashed curve shows the plasmon resonance wavelength shift as a function of the diameter and representative extinction spectra for D = 40 nm and D = 60 nm, respectively. Inset shows the electric field intensity distribution at the plasmon resonance wavelength for D = 40 nm and D = 60 nm, repectively. A substantial increase of the diameter in constant volume apparently contributes to an increase of the extinction intensity as well as a red-shift of the plasmon resonance wavelength.

## References

[b1] Camden J. P. *et al.* Probing the structure of single-molecule surface-enhanced Raman scattering hot spots. J Am Chem Soc. 130, 12616–12617 (2008). 18761451 10.1021/ja8051427

[b2] Kleinman S. L. *et al.* Creating, characterizing, and controlling chemistry with SERS hot spots. Physical Chemistry Chemical Physics. 15, 21–36 (2013). 23042160 10.1039/c2cp42598j

[b3] Stiles P. L., Dieringer J. A., Shah N. C. & Van Duyne R. R. Surface-Enhanced Raman Spectroscopy. Annu Rev Anal Chem. 1, 601–626 (2008). 10.1146/annurev.anchem.1.031207.112814 20636091

[b4] Nie S. M. & Emery S. R. Probing single molecules and single nanoparticles by surface-enhanced Raman scattering. Science. 275, 1102–1106 (1997). 9027306 10.1126/science.275.5303.1102

[b5] Kneipp K. *et al.* Single molecule detection using surface-enhanced Raman scattering (SERS). Phys Rev Lett. 78, 1667–1670 (1997).

[b6] Etchegoin P. G. & Le Ru E. C. A perspective on single molecule SERS: current status and future challenges. Physical chemistry chemical physics: PCCP. 10, 6079–6089 (2008). 18846295 10.1039/b809196j

[b7] Le Ru E. C., Etchegoin P. G. & Meyer M. Enhancement factor distribution around a single surface-enhanced Raman scattering hot spot and its relation to single molecule detection. J Chem Phys. 125, 204701–204713 (2006). 17144717 10.1063/1.2390694

[b8] Fang Y., Seong N. H. & Dlott D. D. Measurement of the distribution of site enhancements in surface-enhanced Raman scattering. Science. 321, 388–392 (2008). 18583578 10.1126/science.1159499

[b9] Markel V. A. *et al.* Near-field optical spectroscopy of individual surface-plasmon modes in colloid clusters. Phys Rev B. 59, 10903–10909 (1999).

[b10] Yue W. S. *et al.* Electron-beam lithography of gold nanostructures for surface-enhanced Raman scattering. J Micromech Microeng. 22, 125007 (2012).

[b11] Haes A. J., Zou S. L. *et al.* Plasmonic materials for surface-enhanced sensing and spectroscopy. Mrs Bull. 30, 368–375 (2005).

[b12] Merk V., Kneipp J. & Leosson K. Gap Size Reduction and Increased SERS Enhancement in Lithographically Patterned Nanoparticle Arrays by Templated Growth. Adv Opt Mater. 1, 313–318 (2013).

[b13] Duan H. G., Hu H. L., Kumar K., Shen Z. X. & Yang J. K. W. Direct and Reliable Patterning of Plasmonic Nanostructures with Sub-10-nm Gaps. Acs Nano. 5, 7593–7600 (2011). 21846105 10.1021/nn2025868

[b14] Zhang H. L., Evans S. D. & Henderson J. R. Spectroscopic ellipsometric evaluation of gold nanoparticle thin films fabricated using layer-by-layer self-assembly. Adv Mater. 15, 531–534 (2003).

[b15] Chang T. W., Gartia M. R., Seo S. J., Hsiao A. & Liu G. L. A wafer-scale backplane-assisted resonating nanoantenna array SERS device created by tunable thermal dewetting nanofabrication. Nanotechnology. 25, 145304 (2014). 24633089 10.1088/0957-4484/25/14/145304

[b16] Musick M. D. *et al.* Electrochemical properties of colloidal Au-based surfaces: Multilayer assemblies and seeded colloid films. Langmuir. 15, 844–850 (1999).

[b17] Brown K. R., Lyon L. A., Fox A. P., Reiss B. D. & Natan M. J. Hydroxylamine seeding of colloidal au nanoparticles. 3. Controlled formation of conductive Au films. Chem Mater. 12, 314–323 (2000).

[b18] Srolovitz D. J. & Goldiner M. G. The Thermodynamics and Kinetics of Film Agglomeration. Jom-J Min Met Mat S. 47, 31–36 (1995).

[b19] Oh Y. J. & Jeong K. H. Glass Nanopillar Arrays with Nanogap-Rich Silver Nanoislands for Highly Intense Surface Enhanced Raman Scattering. Adv Mater. 24, 2234–2237 (2012). 22454295 10.1002/adma.201104696

[b20] Oates T. W. H., Sugime H. & Noda S. Combinatorial Surface-Enhanced Raman Spectroscopy and Spectroscopic Ellipsometry of Silver Island Films. J Phys Chem C. 113, 4820–4828 (2009).

[b21] Leem J. W., Yeh Y. & Yu J. S. Enhanced transmittance and hydrophilicity of nanostructured glass substrates with antireflective properties using disordered gold nanopatterns. Opt Express. 20, 4056–4066 (2012). 22418164 10.1364/OE.20.004056

[b22] Kelly K. L., Coronado E., Zhao L. L. & Schatz G. C. The optical properties of metal nanoparticles: The influence of size, shape, and dielectric environment. J Phys Chem B. 107, 668–677 (2003).

[b23] Kang M., Kim J. J., Oh Y. J., Park S. G. & Jeong K. H. A deformable nanoplasmonic membrane reveals universal correlations between plasmon resonance and surface enhanced Raman scattering. Adv Mater. 26, 4510–4514 (2014). 24668875 10.1002/adma.201305950

[b24] Sun X. & Li H. Gold nanoisland arrays by repeated deposition and post-deposition annealing for surface-enhanced Raman spectroscopy. Nanotechnology. 24, 355706 (2013). 23942082 10.1088/0957-4484/24/35/355706

[b25] Levine J. R., Cohen J. B. & Chung Y. W. Thin film island growth kinetics: a grazing incidence small angle X-ray scattering study of gold on glass. Surf Sci. 248, 215–224 (1991).

[b26] Jiran E. & Thompson C. V. Capillary Instabilities in Thin-Films. J Electron Mater. 19, 1153–1160 (1990).

[b27] Wang D., Ji R. & Schaaf P. Formation of precise 2D Au particle arrays via thermally induced dewetting on pre-patterned substrates. Beilstein J Nanotech. 2, 318–326 (2011). 10.3762/bjnano.2.37 PMC3148046 21977445

[b28] Gunnarsson L., Bjerneld E. J., Xu H., Petronis S., Kasemo B. & Kall M. Interparticle coupling effects in nanofabricated substrates for surface-enhanced Raman scattering. Appl Phys Lett. 78, 802–804 (2001).

[b29] Yu Q., Zhao S., Ye F. & Li S. Determination of octopamine in human plasma by capillary electrophoresis with optical fiber light-emitting diode-induced fluorescence detection. Anal Biochem. 369, 187–191 (2007). 17632069 10.1016/j.ab.2007.06.014

[b30] Manghani K. K., Lunzer M. R., Billing B. H. & Sherlock S. Urinary and serum octopamine in patients with portal-systemic encephalopathy. Lancet. 2, 943–946 (1975). 53430 10.1016/s0140-6736(75)90359-1

[b31] Boulton A. A. Trace amines and mental disorders. Can J Neurol Sci. 7, 261–263 (1980). 7004611 10.1017/s0317167100023313

[b32] Kinniburgh D. W. & Boyd N. D. Determination of plasma octopamine and its level in Renal-disease. Clin Biochem. 12, 27–32 (1979). 376184 10.1016/s0009-9120(79)90048-1

[b33] Whelan C. M., Smyth M. R. & Barnes C. J. HREELS, XPS, and electrochemical study of benzenethiol adsorption on Au(111). Langmuir. 15, 116–126 (1999).

[b34] Wan L. J., Terashima M., Noda H. & Osawa M. Molecular orientation and ordered structure of benzenethiol adsorbed on gold(111). J Phys Chem B. 104, 3563–3569 (2000).

