# Supplementary material for: Repeated Solid-state Dewetting of Thin Gold Films for Nanogap-rich Plasmonic Nanoislands
Source: Sci Rep. 2015 Oct 15;5:14790. doi: 10.1038/srep14790 (PMC4606563; doi:10.1038/srep14790)
Supplement: Supplementary Information [file srep14790-s1.pdf]

Supplementary Information

Repeated Solid-state Dewetting of Thin Gold Films for Nanogap-rich Plasmonic Nanoislands

*Minhee Kang, Sang-Gil Park and Ki-Hun Jeong**

Department of Bio and Brain Engineering and KAIST Institute for Optical Science and Technology, Korea Advanced Institute of Science and Technology (KAIST), 291 Daehak-ro, Yuseong-gu, Daejeon 305-701, Republic of Korea

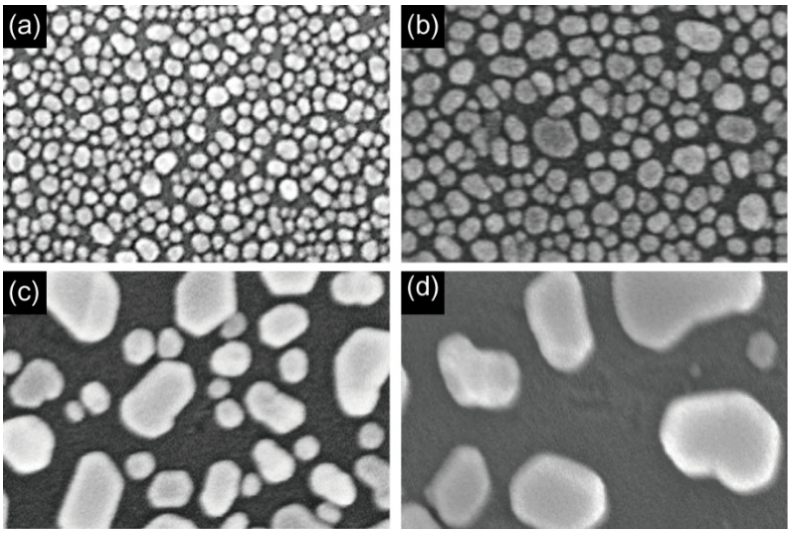

Figure S1. SEM images of Au nanoislands corresponding initial film thickness after a single-step solid-state dewetting process; (a) 5 nm, (b) 7 nm, (c) 10 nm and (d) 14 nm. As the film thickness increases, both the average nanoisland size and the interstitial gap spacing simultaneously increase after a single-step solid-state dewetting process. (image size : 600 x 400 nm)

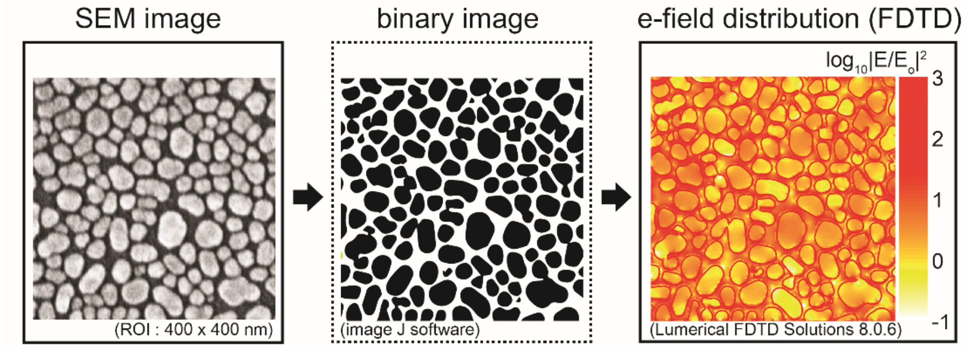

Figure S2. SEM image converts to binary segments for FDTD simulation. Calculated electric field intensity distribution from imported binary image.

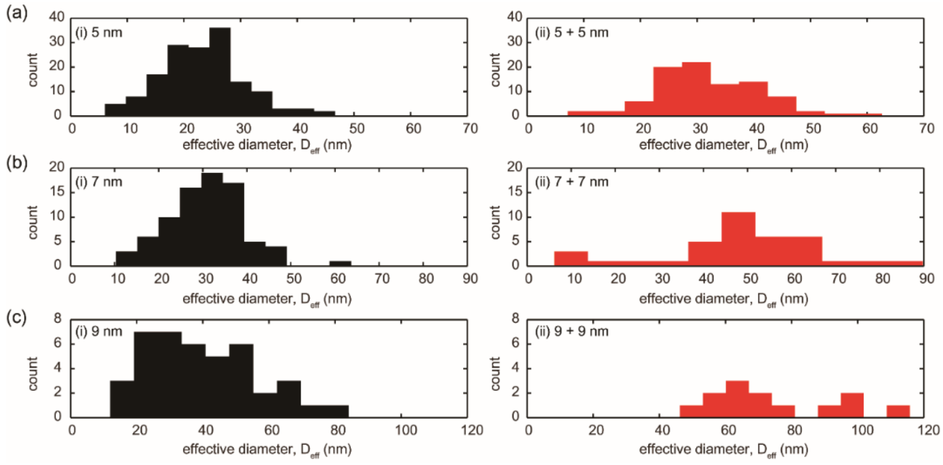

Figure S3. Controlled size and distributions of Au nanoislands for a single-step and repeated dewetting, representative Au thickness of (a) 5 nm, (b) 7 nm, and (c) 9 nm.

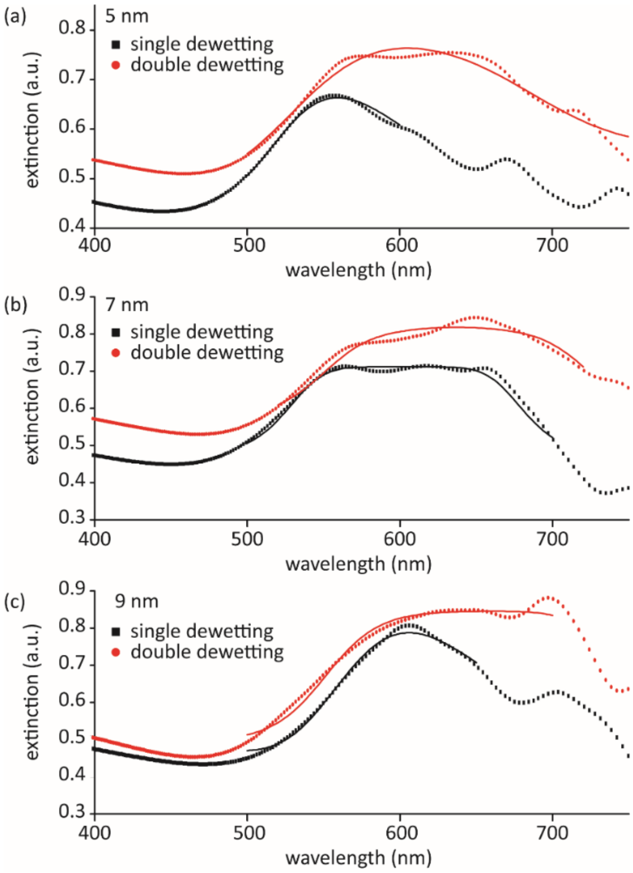

Figure S4. The calculated plasmon resonance wavelength of Au nanoislands extracted from th e SEM image for a single-step dewetting and repeated dewetting with double dewetting, *i.e.,* repeated dewetting with the same thickness, representative Au thickness of (a) 5 nm, (b) 7 nm, and (c) 9 nm.

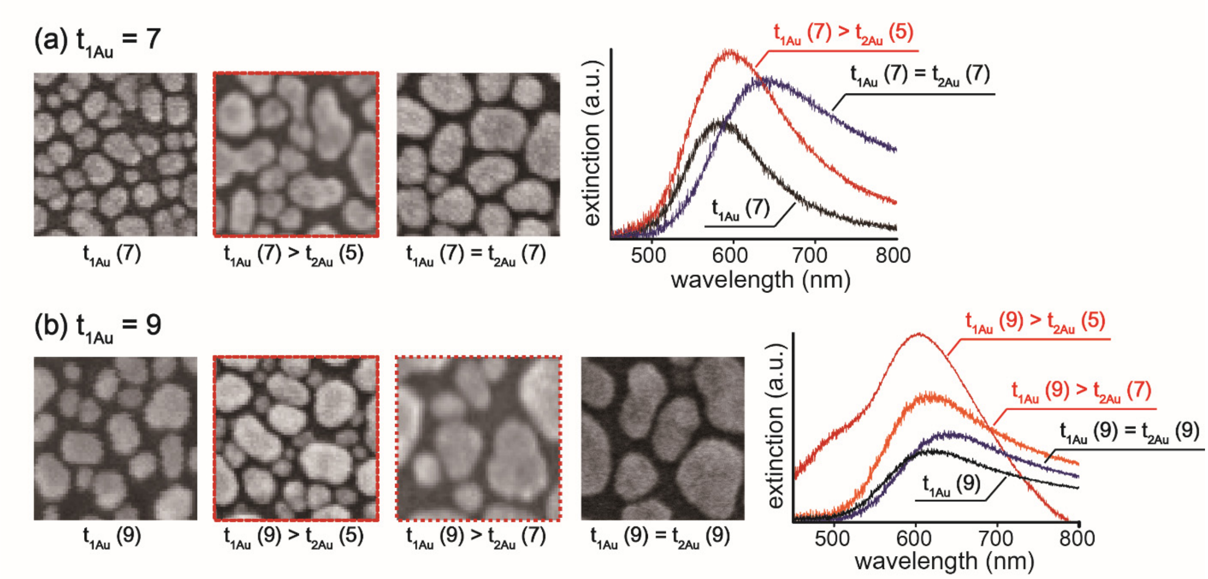

Figure S5. SEM images and the extinction spectra of a single-step and repeated solid-state dewetting of thin Au film. Note that t_{1Au} represents Au film thickness for the first dewetting process and t_{2Au} represents addtional Au film thickness for the second dewetting process. If a given Au thickness for the first dewetting process is thicker than that of additional film for the second dewetting process, the thinner additional film for the second dewetting process may give rise to multiscale nanoislands. The thinner additional film thickness is also responsible for strong plasmonic extinction due to the size difference of Au nanoislands.

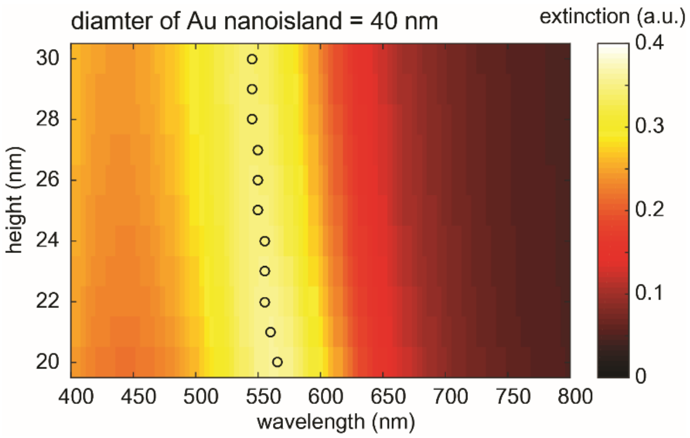

Figure S6. The extinction and plasmon resonance shift depending on the height of nanoislands; the dot shows the plasmon resonance wavelength for Au nanoisland with 40 nm in diameter. The height of Au nanoisland varies from 20 nm to 30 nm. As the height of nanoislands increases, the plasmon resonance wavelengths show little change
